# Emergency Physicians’ Views of Direct Notification of Laboratory and Radiology Results to Patients Using the Internet: A Multisite Survey

**DOI:** 10.2196/jmir.3721

**Published:** 2015-03-04

**Authors:** Joanne Callen, Traber Davis Giardina, Hardeep Singh, Ling Li, Richard Paoloni, Andrew Georgiou, William B Runciman, Johanna I Westbrook

**Affiliations:** ^1^Centre for Health Systems and Safety ResearchAustralian Institute of Health InnovationMacquarie UniversitySydneyAustralia; ^2^Houston VA HSR&D Center for Innovations in Quality, Effectiveness and SafetyMichael E DeBakey Veterans Affairs Medical Center, Department of MedicineBaylor College of MedicineHouston, TXUnited States; ^3^Emergency DepartmentConcord Repatriation and General HospitalSydneyAustralia; ^4^The Joanna Briggs InstituteDepartment of Anaesthesia and Intensive CareUniversity of AdelaideAdelaideAustralia

**Keywords:** Internet, patient safety, electronic health records, patient empowerment, diagnostic tests, emergency care, radiology

## Abstract

**Background:**

Patients are increasingly using the Internet to communicate with health care providers and access general and personal health information. Missed test results have been identified as a critical safety issue with studies showing up to 75% of tests for emergency department (ED) patients not being followed-up. One strategy that could reduce the likelihood of important results being missed is for ED patients to have direct access to their test results. This could be achieved electronically using a patient portal tied to the hospital’s electronic medical record or accessed from the relevant laboratory information system. Patients have expressed interest in accessing test results directly, but there have been no reported studies on emergency physicians’ opinions.

**Objective:**

The aim was to explore emergency physicians’ current practices of test result notification and attitudes to direct patient notification of clinically significant abnormal and normal test results.

**Methods:**

A cross-sectional survey was self-administered by senior emergency physicians (site A: n=50; site B: n=39) at 2 large public metropolitan teaching hospitals in Australia. Outcome measures included current practices for notification of results (timing, methods, and responsibilities) and concerns with direct notification.

**Results:**

The response rate was 69% (61/89). More than half of the emergency physicians (54%, 33/61) were uncomfortable with patients receiving direct notification of abnormal test results. A similar proportion (57%, 35/61) was comfortable with direct notification of normal test results. Physicians were more likely to agree with direct notification of normal test results if they believed it would reduce their workload (OR 5.72, 95% CI 1.14-39.76). Main concerns were that patients could be anxious (85%, 52/61), confused (92%, 56/61), and lacking in the necessary expertise to interpret their results (90%, 55/61).

**Conclusions:**

Although patients’ direct access to test results could serve as a safety net reducing the likelihood of abnormal results being missed, emergency physicians’ concerns need further exploration: which results are suitable and the timing and method of direct release to patients. Methods of access, including secure Web-based patient portals with drill-down facilities providing test descriptions and result interpretations, or laboratories sending results directly to patients, need evaluation to ensure patient safety is not compromised and the processes fit with ED clinician and laboratory work practices and patient needs.

## Introduction

Information and communication technology (ICT) is an essential component for facilitating communication between health professionals within and across health care settings [1,2]. Patients are also increasingly using the Internet to communicate with health care providers and access general and personal health information using email and patient portals tied to hospital-based electronic medical records [3-10]. Given the increasing use of ICT to assist communication, any investigation of suboptimal communication between patients and providers should explore ways in which the Internet could improve information exchange. Breakdown in communication has been identified as a common problem with test result follow-up [11-15]. In the emergency department (ED), up to 75% of test results are missed and the potential impact on patient outcomes includes missed cancer diagnoses [16].

More systematic processes for test result follow-up are needed, including the use of electronic information systems [13,17-20]. It has also been proposed that patient involvement could assist in mitigating missed test results [21-23]. Patients could act as a safety net and be directly notified of their results by the treating clinician transmitting them by email, text messaging (short message service, SMS), letter, or telephone. Alternatively, patients could access test results electronically directly from the laboratory or via a patient portal tied to their hospital electronic medical record (EMR). Physicians would still play a major role in test follow-up and action regarding further treatments, etc. There are moves toward legislating direct notification of tests results in the United States. Since 2004, the Mammography Quality Standards Act has required that a summary of the mammogram report, written in layperson terms, be sent directly to the patient within 30 days [24]. In February 2014, the US Department of Health and Human Services issued a regulation specifying that laboratories must release test reports directly to patients if requested [25].

Studies have shown that patients support direct notification of normal and abnormal test results [5,21,26-29]. However, studies that have explored physicians’ views have only included primary care practitioners [30-36]. Because the ED is a critical area where results are missed, it is important to gauge physicians’ views on direct notification of test results to patients. There are no published studies of emergency physicians’ opinions of patients’ direct access to test results.

The aim of this study was to explore emergency physicians’ current practices of abnormal test result notification and their attitudes to direct patient notification of clinically significant abnormal and normal test results.

Our research questions were 2-fold:

1. What are emergency physicians’ current practices for patient notification of abnormal test results in terms of methods, timing of, and responsibilities for notification?
2. What are emergency physicians’ views about direct patient notification of normal and abnormal test results?

## Methods

### Study Design, Setting, and Population

A cross-sectional paper-based survey was administered to all senior emergency physicians (site A: n=50; site B: n=39) in the EDs of 2 large public metropolitan teaching hospitals in Australia (Table 1). The sites were selected using convenience sampling. Senior emergency physicians were defined as department directors, specialists in emergency medicine, registrars, and senior medical officers and were included based on their involvement and responsibility in relation to test ordering and test result follow-up. The study was approved by the ethics committees from both hospital study sites and the University of New South Wales, Sydney, Australia.

**Table 1:** Characteristics of 2 hospital emergency department (ED) sites.

Characteristics	Hospital site	
	A	B
Hospital beds, n	758	543
Annual inpatient discharges, n	83,898	45,055
Annual ED attendances, n	61,939	35,687
Annual ED discharges home, n	40,713	23,019
Hospitalizations for which the ED is responsible, %	25%	28%
ED admission rates, %	34%	36%

### Survey Instrument

A survey consisting of 22 closed-ended questions was developed based on literature related to patients’ and physicians’ access to clinical information [34,37]. We pilot-tested the survey face-to-face with 2 senior hospital specialists (1 ED physician and 1 rheumatology specialist) from Australia and 10 US primary care practitioners. Based on their feedback, modifications were made to improve clarity of the survey. Fifteen of the survey questions had 7-point Likert response scales with options ranging from strongly agree to strongly disagree.

A detailed description of the design and development of the survey instrument is included in the paper on primary care physicians’ attitudes to directly releasing results to patients [38]. An identical survey was used in both studies. Questions included: current practices and institutional policies for patient notification of abnormal test results (timing, methods, and responsibilities), attitudes toward direct notification of normal and abnormal results, possible physician concerns including lack of patient expertise in interpreting results, previous experiences with missed abnormal test results by themselves or others, patient care decision-making attitudes, and participant demographic details.

For the purposes of the study, direct notification was defined as the automatic release of test results directly to the patient regardless of whether or not the ordering physician had reviewed the results. Direct notification could be achieved by mail, telephone, fax, SMS text message, or by patients accessing their EMR using a patient portal. *Abnormal test results* were defined for this study as clinically significant abnormal results. This includes those that are not immediately life-threatening but required short-term follow-up; for example, newly elevated glucose blood levels or a chest x-ray with a new shadow.

### Data Collection

The survey was self-administered by emergency physicians at the 2 study hospitals between July 1 and September 30, 2012. The survey took each physician approximately 15 minutes to complete. After obtaining staff rosters, researchers approached each ED physician from the study population and asked them to participate in the study. Each physician was informed of the confidential nature of the results and each survey contained a participant information sheet outlining the nature of the study and that completion of the survey implied consent. The survey was left with the ED physician and they were asked to place the completed survey in a dedicated secure box in the ED secretary’s office. The researchers returned at the end of each week to collect the completed surveys and follow-up with nonrespondents. Those physicians who had failed to complete the survey were reminded and given another survey if the previous one had been mislaid.

### Statistical Analysis

Data were analyzed using SPSS version 21.0 (IBM Corp, Armonk, NY, USA) and descriptive statistics calculated. For ease of interpretation, we recoded the dependent variable responses into dichotomous categories. For descriptive analyses and logistic regression, the responses of agree, moderately agree, or strongly agree and disagree, moderately disagree, and strongly disagree were collapsed into agree and disagree, respectively. The category of neither agree nor disagree was retained for descriptive analyses.

We used logistic regression to identify predictors related to emergency physicians’ comfort with direct notification of abnormal test results to patients and their agreement with direct notification of normal test results. Exact methods for logistic regression models were adopted because the sample size of the study was too small for asymptotic methods [39]. The category of neither agree nor disagree was excluded for logistic regression because the number of physicians who responded to this category was small and exact analysis for an ordinal logistic regression model was not available computationally. Two exact logistic regression models were developed using a stepwise forward selection of covariate method [39]. To avoid overfitting the models, we included no more than 2 explanatory variables in each model [40]. We included only variables that were clinically relevant as possible predictors. Predictors in the final models were considered significant at the *P*<.05 level. Odds ratios and their 95% confidence intervals were presented for the predictors included in the final models. The regression analysis was generated using SAS/STAT version 9.3 (SAS Institute, Inc, Cary, NC, USA).

## Results

### Characteristics of Respondents

A total of 89 emergency physicians were invited to complete the survey and 61 complied giving an overall response rate of 69% (61/89) (Table 2). The response rate for hospital A was 74% (37/50) and for hospital B was 62% (24/39).

**Table 2:** Characteristics of emergency physicians from the 2 study hospitals (N=61).

Participant characteristics		Total, n (%)
**Gender**		
	Female	30 (49)
	Male	31 (51)
**Age (years)**		
	20-29	20 (33)
	30-39	17 (28)
	40-49	16 (26)
	50-59	5 (8)
	60-69	3 (5)
**Position** ^a^		
	Senior emergency physician	21 (34)
	Registrar	25 (41)
	SRMO	15 (25)
**Years in practice**		
	<5	25 (41)
	5-10	12 (20)
	11-15	10 (16)
	16-20	4 (7)
	>21	10 (16)

^a^ Senior emergency physicians are board certified specialists in emergency medicine; registrars and senior resident medical officers (SRMOs) are physicians in senior fellowship or residency positions.

### Emergency Physicians’ Attitudes Toward and Concerns About Direct Notification of Test Results

Approximately half of the emergency physicians (54%, 33/61) were not comfortable with patients receiving direct notification of abnormal results, although a similar proportion (57%, 35/61) agreed with direct notification of normal results (Table 3). Most (57%, 35/61) agreed that overall a direct notification system would reduce the number of patients lost to follow-up. Physicians’ major concerns relating to direct notification of abnormal results were confusion (92%, 56/61), lack of expertise necessary to interpret test results (90%, 55/61), and patient anxiety (85%, 52/61). Most also expressed concerns that the patient may seek information that could be unreliable (57%, 35/61). Most physicians were not concerned that direct notification would interfere with the practice of medicine (90%, 55/61) or increase their workload (92%, 56/61). The majority of physicians disagreed with releasing abnormal results directly to patients for human immunodeficiency virus (HIV) (84%, 51/61), cancer screening (84%, 51/61), and chest x-rays (59%, 36/61). Tests which most thought suitable for direct notification were lipid profile (64%), blood glucose (59%), and urinalysis (57%). Approximately half the respondents felt comfortable with releasing abnormal complete blood counts (49%, 30/61), thyroid function tests (48%, 29/61), and electrolytes (44%, 27/61) directly to patients (Table 3).

### Emergency Physicians’ Current Practices and Responsibilities for Direct Notification of Abnormal Results

Most respondents agreed (75%, 46/61) that there were standard policies and procedures for notification of abnormal results in their EDs (Table 4). However, there were mixed responses regarding who should be responsible for notifying patients of results with some physicians (43%, 26/60) stating that it was not always clear who should notify patients of abnormal results. The majority agreed that the ordering physician (or their assigned delegate) should be solely responsible for notifying patients (65%, 39/60) and the majority also agreed that the primary care provider should be responsible for following up abnormal results regardless of who ordered the test (65%, 39/60). Emergency physicians’ practices in relation to methods of patient notification of abnormal test results varied with respondents stating that they always (47%, 25/53) or sometimes (43%, 23/53) phoned the patient. The majority of emergency physicians indicated that they never emailed (100%, 44/44), never faxed (91%, 40/44), and never waited for the patient to contact them (91%, 40/44) regarding abnormal results. In relation to decision making, most respondents (58%, 35/60) thought that they shared responsibility with the patient for deciding on treatments.

**Table 3:** Emergency physicians’ attitudes toward direct notification.

Opinions and concerns about direct notification of test results to patients		Scale, n (%)			n (%)
		Agree/yes	Neither agree or disagree	Disagree/no	
**Attitudes to direct notification of test results to patients (n=61)**					
	I am comfortable with patients receiving direct notification of abnormal test results	24 (39)	4 (7)	33 (54)	
	Do you agree that there should be direct patient notification of normal results?	35 (57)	8 (13)	18 (30)	
	Overall, a direct notification system would reduce the number of patients lost to follow-up	35 (57)	13 (21)	13 (21)	
	Overall, a direct notification system would reduce physician workload	19 (32)	15 (25)	26 (43)	
**Concerns regarding direct notification of abnormal test results** ^a^ **to patients (n=61)**					
	Patient anxiety about test results	52 (85)		9 (15)	
	Patient confusion about test results	56 (92)		5 (8)	
	Patients lack expertise necessary to interpret the results	55 (90)		6 (10)	
	Patient may seek unreliable information	35 (57)		26 (43)	
	Patient may seek care without consulting their primary care provider	29 (48)		32 (53)	
	Interferes with the practice of medicine	6 (10)		55 (90)	
	Physician workload increase	5 (8)		56 (92)	
	I have no concerns	2 (3)		59 (97)	
**If direct notification became the norm, which abnormal test results** ^a^ **would you be comfortable with releasing directly to patients (n=61)**					
	Complete blood count	30 (49)		31 (51)	
	Electrolyte panel	27 (44)		34 (56)	
	Blood glucose	36 (59)		25 (41)	
	Chest x-ray	25 (41)		36 (59)	
	Lipid profile (TC, HDL, LDL, TG)	39 (64)		22 (36)	
	Thyroid blood tests (TSH, T4, TPO)	29 (48)		32 (53)	
	HIV	10 (16)		51 (84)	
	Urinalysis	35 (57)		26 (43)	
	Cancer screening tests (eg, mammography, PAP smear)	10 (16)		51 (84)	
**Please specify at what time interval, after the result became available, would you be comfortable with direct notification of abnormal test results** ^a^ **to patients (n=60)** ^b^					
	24 hours				31 (52)
	48 hours				16 (27)
	7 days				5 (8)
	14 days				0 (0)
	30 days				1 (2)
	Other				7 (12)

^a^ Abnormal test results are clinically significant abnormal results such as newly elevated blood glucose or chest x-ray with new shadow.

^b^ One missing response.

**Table 4:** Emergency physicians’ current practices and responsibilities for direct notification of abnormal results.^a^

Emergency physicians’ current practices and responsibilities for direct notification of abnormal results		n (%)	Scale, n (%)		
			Always	Sometimes	Never
**As part of your usual practice when do you (or staff delegated by you) typically notify patients of abnormal test results? (n=61)**					
	<24 hours	50 (82)			
	24 hours-1 week	11 (18)			
	>1 week	0 (0)			
**In my practice, there are standardized policies and procedures for notification of abnormal test results (n=61)**					
	Agree	46 (75)			
	Neither agree nor disagree	7 (12)			
	Disagree	8 (13)			
**The physician who ordered the test or their assigned delegate should be solely responsible for notifying patients of abnormal test results (n=60)**					
	Agree	39 (65)			
	Neither agree nor disagree	0 (0)			
	Disagree	21 (35)			
**The assigned primary care provider for the care of the patient should always be responsible for following up abnormal test results regardless of who ordered the test (n=60)**					
	Agree	39 (65)			
	Neither agree nor disagree	2 (3)			
	Disagree	19 (32)			
**It is not always clear who should notify patients of abnormal test results (n=60)**					
	Agree	26 (43)			
	Neither agree nor disagree	9 (15)			
	Disagree	25 (42)			
**When it comes to make decision about my patients’ care, I am most comfortable when (n=60)**					
	I make the decision about treatment	0 (0)			
	I make the final decision but consider the patient’s opinion	22 (37)			
	The patient and I share responsibility for deciding treatment	35 (5)			
	The patient makes the final decision but considers my opinion	3 (5)			
	The patient makes the final selection with little input from me	0 (0)			
**Once you have seen an abnormal test result how do you (or staff delegated by you) notify patients?** ^b^					
	Phone		25 (47)	23 (4)	5 (9)
	Email		0 (0)	0 (0)	44 (100)
	Fax		1 (2)	3 (7)	40 (91)
	Letter		3 (7)	23 (50)	20 (44)
	Wait until next appointment		0 (0)	5 (12)	38 (88)
	Schedule a follow-up appointment		3 (7)	14 (32)	27 (61)
	Wait for the patient to contact you		1 (2)	3 (7)	40 (91)

^a^ Abnormal test results are clinically significant abnormal results such as newly elevated blood glucose or chest x-ray with new shadow.

^b^ Not all respondents answered each question.

### Emergency Physicians’ Experiences With Missed Test Results

In the previous 12 months, 21% (13/61) of emergency physicians said they had missed an abnormal result which led to delayed patient care, although half said they did not know whether they had missed a result or not (51%, 31/61). When asked if their colleagues had missed an abnormal result, a higher proportion responded yes (44%, 27/61). Participants reported radiology results to be the most frequently missed (38%, 23/61) followed by microbiology (13%, 8/61) and chemistry (13%, 8/61).

### Results From Exact Logistic Regression Models

The final exact logistic regression model showed that emergency physicians who were not concerned about whether patients might seek unreliable information were 4 times more likely to be comfortable with direct notification of abnormal test results than those who were not concerned about this issue (OR 4.56, 95% CI 1.04-24.30). If direct notification became the norm, physicians would feel more comfortable with releasing abnormal test results on blood glucose than on other test results (OR 15.74, 95% CI 2.84-171.16).

Emergency physicians’ agreement on whether a direct notification system would reduce physician workload was the main factor associated with agreement on direct patient notification of normal test results. Those who agreed that a direct notification system would reduce physician workload were more likely to agree with a direct patient notification of normal results than those who disagreed that it would reduce workloads (OR 5.72, 95% CI 1.14-39.76). Those who were neutral about this issue were more likely to agree on direction notification than those who disagreed, but this difference was not statistically significant (OR 3.84, 95% CI 0.72-27.81).

**Table 5:** Logistic regression exploring predictors of emergency physician comfort with direct patient notification of abnormal test results and agreement with patient notification of normal test results.

Parameter		OR (95% CI)	*P*
**Feel comfortable with patients receiving direct notification of abnormal test results^a^**			
	Concerned that patients may seek unreliable information		.04
	No (reference)	4.56 (1.04-24.30)	
	Yes		
**Feel comfortable with releasing abnormal test results on blood glucose, if direct notification became the norm**			<.001
	Yes	15.74 (2.84-171.16)	
	No (reference)		
	Agree that there should be direct patient notification of normal results		
**Overall, a direct notification system would reduce physician workload**			.03
	Agree	5.72 (1.14-39.76)	.003
	Neutral	3.84 (0.72-27.81)	.14
	Disagree (reference)		

^a^ Abnormal test results are clinically significant abnormal results such as newly elevated blood glucose or chest x-ray with new shadow.

## Discussion

### Principal Results

Although most emergency physicians in our study thought that a direct test notification system would reduce the number of patients lost to follow-up, just over half did not support direct notification of abnormal test results. The main concerns expressed were that it would result in patient anxiety and confusion, and that patients lacked the necessary expertise to interpret test results. Physicians were more likely to be comfortable with direct notification of abnormal test results if they were not concerned about whether patients might seek potentially unreliable information. However, the majority of emergency physicians in our study were comfortable with direct notification of normal test results and they were more likely to be supportive if they thought this would reduce their workload. Approximately 1 in 5 respondents reported missing results in the previous 12 months, whereas half did not know whether they had or not. Radiology results were cited as the most frequently missed. There were diverse responses regarding who should be responsible for notifying patients of abnormal results with a number of respondents stating that this was not always clear.

Two key results from our study, which have not been previously published, focus attention on the unique ED environment, where physicians normally have no continuing relationship with their patients. Most respondents in our study notified patients of abnormal test results via telephone, sometimes they used a letter, and email was never used. ED physicians also showed mixed views regarding who was responsible for notifying the patient of an abnormal result. Lines of responsibility for test notification seem unclear because the majority of respondents in our study thought the physician who ordered the test should be responsible, but the majority also agreed that the primary care provider was responsible as well. These new findings underscore the need to design test notification systems to suit the ED context.

### Limitations

The data were collected from senior emergency physicians from 2 EDs. Emergency physicians from regional and smaller hospitals and junior emergency physicians may have different attitudes. We did not include a question in the survey about the possible responsibility of laboratories (pathology or imaging) with regard to notifying patients of test results, which meant we did not garner emergency physicians’ opinions on this. Qualitative interviews with emergency physicians, laboratory personnel, and information technology staff could have elicited richer information on barriers and facilitators to patients having direct access to their test results.

### Comparison With Prior Work

#### Overview

Studies have shown that patients overwhelmingly support direct notification of their test results [5,9,21,26-29] and when given access to their hospital medical records via a patient portal, test results are often cited as the most frequently accessed information [41-43]. One study surveyed 304 ED patients regarding their use of the Internet and found that the majority were willing to view laboratory results via the Internet [44]. However, when physicians are asked their views of direct notification, they often perceive barriers [5,32,34,36,45]. In contrast to the generally negative opinions from surveys and qualitative studies asking physicians for their views, pilot studies trialing patients’ electronic access to their EMR have reported positive attitudes from physicians [30,31,33,35,46,47]. An example is the positive findings reported from the Open Notes project [31,37,48].

#### Physicians’ Concerns About Patients’ Anxiety if Results Are Directly Notified

Physicians’ perceived concerns about direct access to test results raising anxiety levels of patients is an often reported issue [32,34,45,49] and was also a finding from our study. However, studies which measured whether patients’ access to medical records and test results caused undue anxiety found no increase in their anxiety levels [23,35,50-52] and have reported positive outcomes for patients. A systematic review to determine the effect of providing patients with access to their medical records, although not specifically reporting on test results, concluded overall that access reduced or had no effect on patient anxiety [53].

#### Physicians’ Concerns About Patients’ Lack of Clinical Knowledge if Results Are Directly Notified

A concern expressed by emergency physicians in our study was that patients lacked the necessary expertise or knowledge to interpret test results; emergency physicians were more likely to be comfortable with release of abnormal results if they were not concerned about patients seeking potentially unreliable information. A pilot study exploring patient access to electronic laboratory results via a patient portal attempted to address this issue by providing easily accessible test result reference information for patients [33]. Each test result had an information button with a hyperlink to general reference information about the result, and they reported that patients in 1 primary practice who viewed results also viewed reference information in 50% of cases [33]. Other studies have reported patients’ lack of understanding and confusion when receiving written histopathology reports following endoscopy [54] and mammography [55], which underscores the need to provide further background/reference information to patients to enhance comprehension of the test result report.

#### Physicians’ Concerns About the Timing of Reporting Results Directly to Patients

Timing of release of information was identified as important in our study with the majority of emergency physicians indicating that they would prefer a 48-hour delay for release of abnormal results to allow them time to contact the patient first. Patients have indicated that timeliness of notification of test results is important [28,29,54,55] and this was taken into account in the Wald et al pilot study [33], as an expert panel of clinicians identified the set of results for direct release and their timing rules (immediate release or a 2-day embargo).

#### Physicians’ Concerns About Direct Notification of Abnormal Results

Our study found whether a result is normal or abnormal is an issue for physicians in relation to reporting test results directly to patients. Most emergency physicians were not comfortable with releasing results that might have a significant emotional impact on patients, such as abnormal HIV, mammography, and PAP smears. This finding is supported by results from a survey of primary care physicians by Sung et al [32], which indicated physicians were significantly more interested in reporting normal rather than abnormal results (*P*<.001). That study found that the level of interest in direct reporting of results declined progressively depending on the perceived emotional impact of the result on the patient, from low (dual energy x-ray absorptiometry scan) to intermediate (genital herpes testing) to high (breast biopsy) (*P*<.001) [32].

#### Physicians’ Concerns Regarding Direct Notification of Radiology Results

Radiology result follow-up remains a vulnerable area [15,56,57]. Although allowing patients direct access to certain radiology results is gaining some support from radiologists [56], some have expressed concerns about imaging results raising patients’ anxiety because the terms used would be unfamiliar and there might be too many requests from patients to meet with radiologists to seek further information and explain the test results [45]. If direct online reporting was instituted, mixed views from physicians and radiologists about how much information patients should have (ie, the full report versus just the conclusions) have been reported [45]. Our study found similar equivocal results in relation to patients’ direct access to radiology reports: the majority were not comfortable with patients directly accessing abnormal chest x-rays (although 41% agreed), and the majority (83%) also did not agree with patients being sent abnormal cancer screening tests such as mammography and PAP smear results. In our study, physicians reported that radiology was the most frequently missed test result; if there is no process of systematic feedback to physicians regarding missed results it is difficult to understand how they learn about test results which they, or a colleague, have missed [58-60].

#### Physicians’ Views Regarding Workload if Results are Directly Notified to Patients

Any intervention introduced into the emergency care context must weigh up the potential impact on the workload of busy emergency clinicians. Our study found that the majority of emergency physicians were not concerned that direct notification of results to patients would increase their workload. In relation to direct notification of normal test results, they were more likely to agree with the process if they believed it would reduce their workload. Other studies have supported this finding [30,32,61].

#### Physicians’ Views Regarding Responsibility for Test Result Follow-Up

Our study showed that there were mixed views regarding responsibility for notifying patients of abnormal results with a lack of clarity regarding the responsibilities of the ordering physician and the primary care provider. Results pending are particularly prevalent for discharged patients from the ED who often have a short stay. Responsibility for follow-up of results, which may or may not be listed on the ED discharge summary, is impacted by unclear lines of responsibility for follow-up between local medical officers and hospital doctors, combined with emergency physicians’ lack of a continuing relationship with the patient [14,19,62,63]. Electronic discharge summaries can play a role in improving information transfer between hospital and community settings. However, critical information can still be missing [64,65]. Others studies have made recommendations for test follow-up responsibility and these need to be assessed to ensure they apply in the ED context [14,66]. The issue of whether patients’ direct access to test results challenges the physicians’ role as an information gatekeeper has been raised by some [30,49]. However, the majority of emergency physicians in our study did not think that direct notification would interfere with the practice of medicine, so concerns regarding physician role adjustments may be overstated.

#### How Emergency Physicians Currently Communicate Abnormal Results to Patients

Our study found that most ED physicians notified patients about abnormal test results by telephone. Other studies have shown that patients prefer a direct phone call from the physician for abnormal results [22,26,29,67]; however, studies of email communications between patients and physicians for result notification have also reported positive experiences from patients [28,68]. Physicians still express some concerns, such as managing clinical issues by email and integrating email into office work processes [4].

### Conclusions

Future work needs to determine if direct notification of test results to patients leads to improved follow-up of abnormal results. Methods of ensuring patients can access test results directly, including secure Web-based patient portals with drill-down capabilities providing test descriptions and result interpretations need to be evaluated in terms of patient outcomes, cost, and patient usability across socioeconomic groups. Implications of patients “pulling” results from a laboratory information system using a patient portal tied to a hospital-based EMR versus test results being “pushed” automatically to patients needs evaluation.

The fast-paced ED environment presents a number of unique challenges for test result follow-up. Although notification of test results to ED patients has promise, it is important to ensure that methods for direct notification suit the environment and work practices of ED clinicians and laboratories and meet patient needs. Efforts should be directed toward establishing a clear set of recommendations regarding which test results should be directly notified to which patients, methods of notification, and the timing of notification. Emergency physicians’ concerns regarding anxiety, confusion, and lack of patients’ expertise to interpret results should be addressed in order to promote wider test results access to patients.

## Abbreviations

ED: emergency department
EMR: electronic medical record
ICT: information and communication technology
